# Public Health Benefits of Applying Evidence-Based Best Practices in Managing Patients Hospitalized for COVID-19

**DOI:** 10.1093/cid/ciae517

**Published:** 2024-10-25

**Authors:** Andre C Kalil, Aastha Chandak, Luke S P Moore, Neera Ahuja, Martin Kolditz, Roman Casciano, Ananth Kadambi, Mohsen Yaghoubi, Sotirios Tsiodras, Jakob J Malin, Essy Mozaffari, Michele Bartoletti

**Affiliations:** Division of Infectious Diseases, University of Nebraska Medical Center, Omaha, Nebraska, USA; Evidence and Access, Certara, New York, New York, USA; Department of Infectious Diseases, Imperial College, London, United Kingdom; Department of Internal Medicine, Stanford University School of Medicine, Stanford, California, USA; Medical Department I, Division of Pulmonology, University Hospital Carl Gustav Carus, TU Dresden, Dresden, Germany; Evidence and Access, Certara, New York, New York, USA; Evidence and Access, Certara, New York, New York, USA; Evidence and Access, Certara, New York, New York, USA; Professor of Medicine and Infectious Diseases, National & Kapodistrian University of Athens Medical School, Chair, 4th Department of Internal Medicine, Attikon University Hospital, Athens, Greece; Department I of Internal Medicine, Division of Infectious Diseases, Faculty of Medicine and University Hospital Cologne, University of Cologne, Cologne, Germany; Medical Affairs, Gilead Sciences, Foster City, California, USA; Department of Biomedical Sciences, Humanitas University, Pieve Emanuele, Milan, Italy; Infectious Disease Unit, IRCCS Humanitas Research Hospital, Milan, Italy

**Keywords:** COVID-19 model, SARS-CoV-2, remdesivir, real world data, mortality, data science, dexamethasone

## Abstract

**Background:**

As coronavirus disease 2019 (COVID-19)–related mortality remains a concern, optimal management of patients hospitalized for COVID-19 continues to evolve. We developed a population model based on real-world evidence to quantify the clinical impact of increased utilization of remdesivir, the effectiveness of which has been well established in hospitalized patients with COVID-19.

**Methods:**

The PINC AI healthcare database records for patients hospitalized for COVID-19 from January to December 2023 were stratified by those treated with or without remdesivir (“RDV” and “no RDV”) and by supplemental oxygen requirements: no supplemental oxygen charges (NSOc), low-flow oxygen (LFO), and high-flow oxygen/non-invasive ventilation. Key vulnerable subgroups such as elderly and immunocompromised patients were also evaluated. The model applied previously published hazard ratios (HRs) to 28-day in-hospital mortality incidence to determine the number of potential lives saved if additional no RDV patients had been treated with remdesivir upon hospital admission.

**Results:**

Of 84 810 hospitalizations for COVID-19 in 2023, 13,233 no RDV patients were similar in terms of characteristics and clinical presentation to the RDV patients. The model predicted that initiation of remdesivir in these patients could have saved 231 lives. Projected nationally, this translates to >800 potential lives saved (95% confidence interval, 469–1126). Eighty-nine percent of potential lives saved were elderly and 19% were immunocompromised individuals. Seventy-six percent were among NSOc or LFO patients.

**Conclusions:**

This public health model underscores the value of initiating remdesivir upon admission in patients hospitalized for COVID-19, in accordance with evidence-based best practices, to minimize lives lost because of severe acute respiratory syndrome coronavirus 2 infection.

## Teaser

In 2023, a substantial number of lives could have been saved in the United States if additional appropriate patients hospitalized for COVID-19 had been treated with remdesivir upon hospital admission, especially in vulnerable populations (elderly, immunocompromised, multiple comorbidities).

Although coronavirus disease 2019 (COVID-19) has shifted from pandemic to endemic status and there is generally reduced interest regarding COVID-19, severe acute respiratory syndrome coronavirus 2 (SARS-CoV-2) infection-related mortality among vulnerable populations remains a concern. The Centers for Disease Control and Prevention (CDC) estimated that there were ∼75 000 COVID-19–related deaths in the United States in 2023 [1] and one of the top 10 causes of mortality in the United States [2]. The recent rise in number of SARS-CoV-2 infections driven by KP.2, KP.2.3, KP.3, LB.1, and KP.3.1.1 variants in the United States [3] and BA.2.86 and JN.1 globally [4] highlights the need for continued management of patients with COVID-19 in a manner consistent with the latest treatment guidelines [5–7], especially in the most vulnerable hospitalized patients, as well as an opportunity to continue improving institutional treatment protocols to reflect the latest evidence.

Clinicians’ understanding of the optimal management approach for patients with COVID-19 continues to evolve [8, 9]. Remdesivir [10] is an antiviral with proven efficacy for reduced time to recovery [11, 12] and improved clinical outcomes for patients hospitalized for COVID-19 [13]. In recent real-world evidence studies, remdesivir was associated with reduced mortality among patients hospitalized for severe COVID-19 [14] regardless of supplemental oxygen requirements upon admission [14, 15]. Furthermore, remdesivir is recommended as the standard of care in the final version of the US National Institutes of Health (NIH) guidelines [7] for patients hospitalized because of COVID-19 and not requiring invasive supplemental oxygen therapy.

In prior extensive assessment of remdesivir's effectiveness using the PINC AI healthcare database (PHD, formerly Premier Healthcare Database) [14–17], a large hospital database in the United States, we noted that substantial number of patients hospitalized for COVID-19 from 2021 onwards may not have been treated in accordance with the latest NIH guidelines, especially vulnerable populations [7, 18–20]. These studies also showed that patients hospitalized for COVID-19 and treated with remdesivir experienced a significantly lower mortality rate than those who were not. Collectively, these insights suggest that, despite the established efficacy of remdesivir in hospitalized patients with SARS-CoV-2 infection [13–15, 17–21], the early-pandemic urgency to reevaluate and modify treatment practices in real time may have lessened. If true, there exists the potential for a substantial positive impact on overall public health because of reduced COVID-19–related mortality if additional appropriate patients hospitalized for COVID-19 receive remdesivir upon admission. However, to our knowledge, this impact has not yet been quantitatively assessed in the United States or elsewhere.

We therefore sought to quantify the potential public health impact if emerging evidence-based best practices on the use of remdesivir for the treatment of patients hospitalized for COVID-19 were more routinely applied in clinical practice.

## METHODS

We developed a model to quantify the number of lives potentially saved if additional appropriate patients hospitalized for COVID-19 in US hospitals in 2023 had been treated with remdesivir upon admission (within 2 days of hospitalization) in accordance with evidence-based best practices and guideline recommendations.

### Data Sources

Three primary sources of data were used in the model:

- Patient-level data for overall 28-day in-hospital mortality (defined as discharge status of “expired” or “hospice”) in the PHD (www.pinc-ai.com) for all patients hospitalized for COVID-19 from January to December 2023.
- Published hazard ratios (HRs) for the relative risk of 28-day in-hospital mortality associated with the initiation of remdesivir upon admission in the PHD population using a propensity score (PS) matching approach [18–20]. A summary of the specific HRs used in the model is provided in Supplementary Tables 1 and 2.
- United States weekly hospitalization rates published by the CDC in its COVID-19 data tracker [22].

### Model Cohort

Patients hospitalized for COVID-19 in PHD were stratified into 2 groups: patients who were treated with remdesivir within the first 2 days of their hospital admission (“RDV”) and those who were not treated with remdesivir at any time during their hospital stay (“no RDV”). The primary focus of the model was to assess the potential impact if remdesivir had been administered to no RDV patients who were similar in terms of their overall characteristics and clinical presentation to RDV patients in the same period. To achieve this, the model applied remdesivir treatment only to the PS-matched no RDV patients used to derive the HR estimates as described previously (“model cohort”) [18–20]. All other patients were excluded from the analysis.

### Model Implementation

The model was developed and programmed in Microsoft Excel with 2 main steps:

- Compute the expected 28-day in-hospital mortality in the PHD population had remdesivir treatment been initiated upon admission in the model cohort: apply the HR representing the estimated 28-day in-hospital mortality benefit of initiation of remdesivir treatment compared to not initiating treatment to the model cohort. (Step 1)
- Determine the total number of lives potentially saved in the PHD population had remdesivir treatment been initiated in the model cohort upon admission: Subtract the result in (1) from the observed 28-day in-hospital mortality incidence in the model cohort. (Step 2)

### Base Case

The base case estimates the number of potential lives saved in 2023 in the PHD population if all matched no RDV patients hospitalized for COVID-19 had received remdesivir upon admission. Model outputs were stratified by several subgroups, including elderly (age ≥65 years), immunocompromised (IC) patients, and patients with Charlson Comorbidity Index (CCI) ≥2, and by 3 categories of baseline supplemental oxygen requirements: no supplemental oxygen charges (NSOc), low-flow oxygen (LFO), and high-flow oxygen/noninvasive ventilation (HFO/NIV). The estimated number of lives saved is reported as a point estimate along with 95% confidence interval (CI) calculated using the upper and lower 95% confidence limits of the associated HR for each stratification.

Subsequently, we performed a national-level projection based on the proportion of all US hospitalizations for COVID-19 represented within the PHD population. Using CDC estimates, there were 724 780 COVID-19–associated hospitalizations in the United States in 2023. The PHD captured 206 366 COVID-19–associated hospitalizations in 2023, representing 28.5% of all US COVID-19–associated hospitalizations. Therefore, a factor of 3.5 (1/0.285) was applied to PHD-level model outputs for national-level projections.

### Alternate Scenarios

Additional scenario analyses were conducted to expand on the base case. Scenario analyses and the rationale for their consideration are summarized in Table 1. For scenarios 1 (overall Omicron period), 4 (remdesivir+dexamethasone [DEX] vs DEX monotherapy) and 5 (elderly subgroup of patients), we applied alternate HRs provided in Supplementary Tables 1 and 2. For scenarios 2 (exclusion of concomitant COVID-19 treatments) and 3 (inclusion of patients on invasive mechanical ventilation), we applied the same HRs as used in the base case to alternate patient populations.

**Table 1.** Alternate Scenarios to Characterize Potential Benefit of Initiating Remdesivir in Patients Hospitalized for COVID-19

Scenarios	Rationale
1. Apply HRs derived from analysis using PHD data from December 2021 to February 2024 (overall Omicron period) to the observed overall mortality	Ensures comprehensive evaluation across different variants of concern (may include some Delta variant)
2. Exclude patients on treatments other than remdesivir (baricitinib, tocilizumab, or oral antiviral) from the model cohort	Given the possibility of confounding, removes any presumption of benefit for remdesivir use in no RDV patients receiving baricitinib, tocilizumab, or oral antivirals
3. Include IMV patients in the model cohort	Highlights the additional potential lives saved with initiation of remdesivir upon admission in patients who are among the most critically ill
4. Evaluate potential benefit of remdesivir in patients initiated on dexamethasone monotherapy to treat COVID-19 (RDV + DEX vs DEX mono)	Alternate to the base case focusing only on no RDV patients who were receiving DEX monotherapy and applying alternate HRs. Patients receiving other COVID-19 treatments were excluded from the matched no RDV patients as described in scenario 2.
5. Conduct age-stratified analyses for the elderly population (65–74, 75–84, and ≥85 years)	Evaluates the impact of increasing age in an already vulnerable population.

Abbreviations: DEX, dexamethasone; HR, hazard ratio; IMV, invasive mechanical ventilation; PHD, PINC AI healthcare database; RDV, remdesivir.

## RESULTS

Demographics of patients hospitalized for COVID-19 are provided in Table 2. Similar demographics for the subgroups are provided in Supplementary Table 3.

**Table 2.** Demographics of the PHD Population Hospitalized for COVID-19 in 2023

		n = 84 810
Age group (y)	<18	3078 (3.6%)
	18–49	4897 (5.8%)
	50–64	11 613 (13.7%)
	≥65	65 222 (76.9%)
Gender, female		44 289 (52.2%)
Race	White	65 510 (77.2%)
	Black	10 461 (12.3%)
	Asian	2224 (2.6%)
	Other	6615 (7.8%)
Ethnicity	Hispanic	7894 (9.3%)
	Non-Hispanic	71 380 (84.2%)
	Unknown	5536 (6.5%)
Hospital size, no. of beds	<100	8550 (10.1%)
	100–199	15 018 (17.7%)
	200–299	15 930 (18.8%)
	300–399	14 861 (17.5%)
	400–499	9536 (11.2%)
	≥500	20 915 (24.7%)
CCI	0	13 182 (15.5%)
	1	18 521 (21.8%)
	2	15 691 (18.5%)
	≥3	37 416 (44.1%)
Comorbid conditions	Obesity	18 271 (21.5%)
	COPD	30 553 (36.0%)
	Cardiovascular disease	73 376 (86.5%)
	Diabetes	30 874 (36.4%)
	Renal disease	24 933 (29.4%)
	IC condition^a^	15 116 (17.8%)
	Cancer	6681 (7.9%)
Baseline supplemental oxygen requirements	NSOc	56 748 (66.9%)
	LFO	17 448 (20.6%)
	HFO/NIV	9035 (10.7%)
	IMV/ECMO	1579 (1.9%)
COVID-19 treatments upon admission	Remdesivir + dexamethasone	26 760 (31.6%)
	Dexamethasone monotherapy	16 980 (20.0%)
	Remdesivir monotherapy	13 640 (16.1%)
	Other (baricitinib, tocilizumab, oral antivirals)	1340 (1.6%)
	None of above COVID-19 treatments	26 090 (30.8%)

Abbreviations: CCI, Charlson Comorbidity Index; COPD, chronic obstructive pulmonary disease; HFO/NIV, high-flow oxygen/noninvasive ventilation; HIV, human immunodeficiency virus; IC, immunocompromised; IMV/ECMO, invasive mechanical ventilation/extracorporeal membrane oxygenation; LFO, low-flow oxygen; NSOc, no supplemental oxygen charges; PHD, PINC AI healthcare database; y, years.

^a^IC conditions include cancer, transplant, hematologic malignancies, immunosuppressive medications, toxic effects of antineoplastics, primary or severe combined immunodeficiencies, asplenia, bone marrow failure/aplastic anemia, or HIV.

Table 3 summarizes the observed hospitalizations, deaths, and remdesivir treatment in the PHD population. In 2023, there were a total of 84 810 hospitalizations for COVID-19 documented in PHD, of which 65 222 were elderly patients (age ≥65 years), 15 116 were IC patients, and 53 107 were patients with CCI ≥2. There were a total of 4676 deaths in this population in 2023. Overall, 48% of patients were treated with remdesivir upon admission (within the first 2 days of hospitalization), a percentage consistent with that observed across all subgroups (49%–52%). Forty-nine percent of patients (41 876) did not receive remdesivir at any point during their admission (no RDV patients).

**Table 3.** Hospitalizations, Deaths and Remdesivir Treatment Among the PHD Population Hospitalized for COVID-19

	Overall	Elderly (age ≥65 y)	IC	CCI ≥2
Total number of patients hospitalized	84810	65 222	15 116	53 107
Observed 28-day in-hospital deaths	4676 (6%)	4254 (7%)	1243 (8%)	3761 (7%)
Patients receiving remdesivir on admission (RDV patients)^a^	40 400 (48%)	32 029 (49%)	7935 (52%)	26 210 (49%)
Patients not receiving remdesivir during hospitalization (no RDV patients)	41 876 (49%)	31 168 (48%)	6520 (43%)	25 045 (47%)

Data are presented as n (%).

Abbreviations: CCI, Charlson Comorbidity Index; IC, immunocompromised; PHD, PINC AI healthcare database; RDV, remdesivir; y, years.

^a^A small number of patients (<5%) received remdesivir later during their hospitalization and are not included.

### Base Case

Table 4 summarizes the base case model outputs, including total patients treated with remdesivir, estimated lives saved in the PHD population and projected nationally, as well as results for the subgroups. In the base case, among the 41 876 no RDV patients, just under one-third were included in the model cohort (13 233 patients) and considered for counterfactual remdesivir treatment. The model estimates that 231 lives would have been potentially saved within the PHD population (5.5% of all observed deaths) if all patients in the model cohort had been treated with remdesivir upon admission.

**Table 4.** Base Case Model Results

	Overall	Elderly (age ≥65 y)	IC	CCI ≥2
PHD Population				
Model cohort^a^	13 233	10 639	2138	9175
Total potential lives saved with remdesivir treatment in the model cohort	231 (133–320)	240 (145–319)	44 (−11–86)	193 (111–268)
NSOc	110 (64–148)	102 (56–142)	8 (−19–29)	90 (52–121)
LFO	64 (36–91)	77 (49–99)	20 (4–33)	55 (31–78)
HFO/NIV	56 (32–80)	60 (38–77)	14 (3–23)	47 (27–68)
National-level projections				
Projected additional patients treated with remdesivir across the United States	46 447	37 342	7504	32 204
Total potential lives saved in the United States in 2023 with additional remdesivir treatment	811 (469–1126)	725 (438–961)	230 (−60–450)	871 (503–1210)

Data are presented as point estimate (95% CI). Negative lower bounds indicate the possibility of remdesivir-induced harm; however, this is observed only in IC patients with small sample size that resulted in wide variability in the estimated HR.

Abbreviations: CCI, Charlson Comorbidity Index; CI, confidence interval; HFO/NIV, high-flow oxygen/noninvasive ventilation; HR, hazard ratio; IC, immunocompromised; LFO, low-flow oxygen; NSOc, no supplemental oxygen charges; PHD, PINC AI Healthcare Database; y, years.

^a^Patients who were propensity score–matched to remdesivir treated patients in the 2023 PHD sample.

The national-level projections suggest that initiation of remdesivir treatment upon admission in a patient cohort with similar characteristics as the model cohort (projected to be 46 447 patients nationally) may have saved between 469 and 1126 hospital-related deaths from COVID-19 across the United States. Approximately 89% of potential lives saved were in elderly patients and 19% in IC patients. Overall, 76% of potential lives saved were patients with NSOc or requiring LFO (∼47% and ∼27%, respectively). Similarly, 74% and 66% of potential lives saved were patients with NSOc or requiring LFO in the elderly and IC populations, respectively.

### Alternate Scenarios

An overview of the rationale for scenarios 1–5 is provided in Table 1.

**Figure 1.**
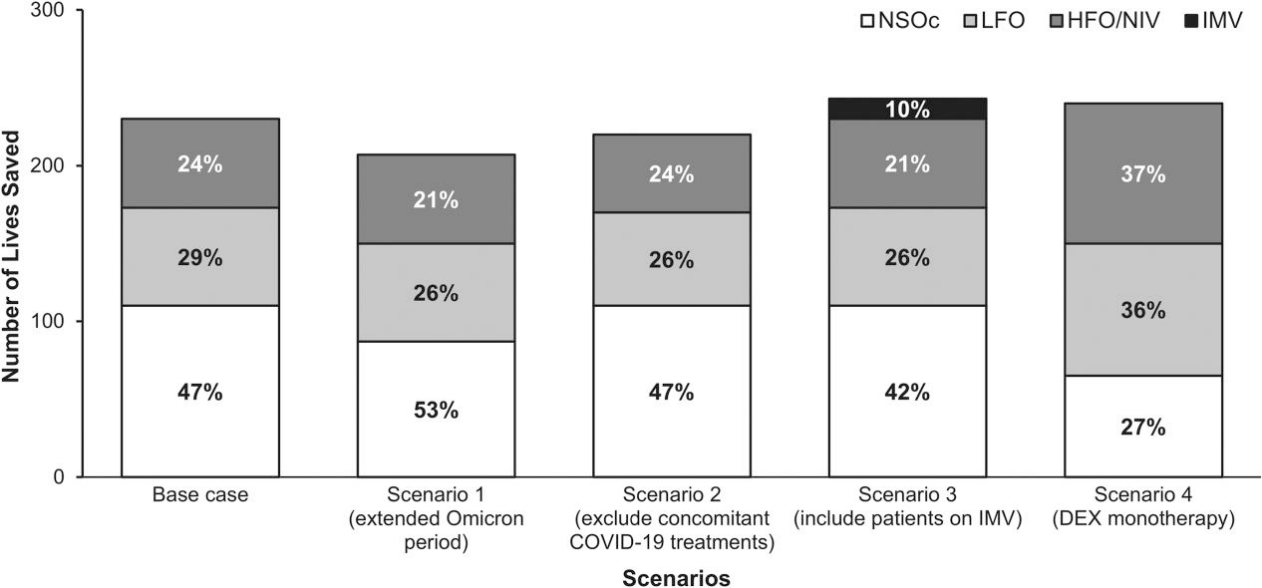
Potential lives saved in the PHD Population hospitalized for COVID-19 if remdesivir were initiated in the model cohort. Base case and scenarios 1–4 results are shown. Stacked bar percentages show relative impact of remdesivir treatment stratified by baseline supplemental oxygen requirements. Scenario 1: apply HR derived from the full omicron period; scenario 2: exclude patients on baricitinib, tocilizumab, or oral antivirals; scenario 3: include patients on IMV; scenario 4: compare RDV + DEX vs DEX monotherapy (excluding prior nonremdesivir treatments). Abbreviations: DEX, dexamethasone; HFO, high-flow oxygen; HR, hazard ratio; IMV, invasive mechanical ventilation; LFO, low-flow oxygen; NIV, noninvasive ventilation; NSOc, no supplemental oxygen charges; PHD, PINC AI healthcare database; RDV, remdesivir.

Most of the scenario 1–4 results were comparable to the base case (Figure 1). Detailed results and discussion of scenario 1 are in Supplementary Table 4; several specific findings for scenarios 2–4 are noteworthy. First, exclusion of patients receiving baricitinib, tocilizumab, or other antivirals from the model cohort (scenario 2) did not substantially affect the percentages or total potential lives saved. Second, inclusion of patients requiring invasive mechanical ventilation (IMV) in the model cohort (scenario 3) added ∼10% to the potential lives saved, indicating the potential survival benefit for initiating remdesivir in hospitalized patients with the highest baseline supplemental oxygen requirement. Finally, when the model evaluated remdesivir + DEX vs DEX monotherapy (scenario 4), patients requiring HFO/NIV accounted for 36% of potential lives saved and patients with NSOc accounted for 27%.

Further stratification of the elderly population (age ≥65 years) showed the largest number of potential lives saved among patients aged 75–84 years, with a proportionally larger impact seen in the NSOc subgroup (scenario 5, Supplementary Figure 1).

## DISCUSSION

The optimal management of patients hospitalized for COVID-19 continues to evolve as the virus itself does, requiring ongoing adaptation of clinical practice to reflect the latest evidence-based research. Recent real-world studies on the outcomes of patients hospitalized for COVID-19 have continued to strengthen the evidence on the effectiveness of early remdesivir treatment across a broad spectrum of patients [14, 15, 17, 18]. Although the NIH and the National Institute of Allergy and Infectious Diseases ACTT-1 (Adaptive COVID Treatment Trial) demonstrated the efficacy of remdesivir in patients hospitalized for COVID-19, neither ACTT-1 nor other early-pandemic randomized controlled trials were designed or powered to measure treatment efficacy in all settings and subgroups of interest [13, 23–25]. The lack of subgroup-specific evidence may have led to clinical underutilization of remdesivir, which persists today in patients who might benefit from treatment with remdesivir.

Our research showed that approximately 49% of patients hospitalized for COVID-19 in the United States did not receive remdesivir as part of their treatment regimen upon admission in 2023. Furthermore, more than 16% of patients not receiving supplemental oxygen received DEX without concomitant remdesivir use upon admission, against NIH guideline recommendations [7]. This pattern of treatment has persisted despite the wide availability of remdesivir in most hospitals and the recommendation for treatment in COVID-19 guidelines for NSOc, LFO, and HFO/NIV patients [5–7]. Perhaps this is a reflection of the inherent complexities faced by clinicians in their choice of treatment for individual patients. It is also possible that legacy restrictions on remdesivir use in local institutional protocols or formulary guidelines based on early-pandemic evidence continue to remain in place. Regardless of the underlying reason, our analysis showed that nearly one-third of patients who did not receive remdesivir upon admission were substantially similar in their characteristics and clinical presentation to those who did receive remdesivir. Initiating treatment in this population is estimated to result in 1 life saved for every 58 patients treated overall and for the most vulnerable populations such as elderly and immunocompromised, 1 life could be saved for every 45 and 48 patients, respectively, treated with remdesivir. At the national level, this translates to a conservative estimate of 469 to 1126 lives saved in 2023, but it is possible that more patients would be appropriate for remdesivir treatment leading to a higher potential lives saved beyond our model projections.

Although appropriate to control for potential bias and confounding between groups, researchers have shown that when using PS matching, inferences cannot be made about the effect of treatment in the entire population of actual patients who are treated by clinicians. Because of this limitation, we have appropriately applied the estimated benefits of remdesivir only to the patient population included in the derivation of the HR estimates (ie, the matched no RDV patients). However, sensitivity analyses presented by Mozaffari et al [19, 20] using inverse probability of treatment weighting suggest that the HR estimates from the PS-matched analyses are broadly applicable across the majority of the hospitalized COVID-19 patient population, and not simply applicable to the less than one-third of untreated patients in the PS-matched group. This implies a substantial upside to the potential lives saved. Given that administration of remdesivir causes no significant adverse events as evidenced by prior research (including randomized controlled trials and real-world studies [10, 26]), its use presents minimal risk and broader appropriate use may be warranted.

Our analysis and projected lives saved are based on a robust, fit-for-purpose data source and conservative analytic approach. We developed our model using the PHD, a large, real-world database, capturing approximately 25% of hospital encounters across the United States [16]. Additionally, extensive peer-reviewed research formed the basis for the HR estimates underpinning the model [14, 17, 19, 20, 27]. Finally, reliable statistics from the US Centres for Disease Control and Prevention were used to project our findings to the US national level [22].

Despite these strengths, there are potential limitations of the model. First, the HRs applied were derived from analysis of a single real-world data source, an approach that, although robust, does not consider available randomized, controlled trial–based evidence on remdesivir in hospitalized patients such as that reported in the recent meta-analysis by Amstuz et al [21], which are based on trials from the early pandemic. While these meta-analytic efficacy results are potentially relevant to our model, findings were reported as odds ratios or relative risk rather than HR, precluding a direct use of these data. However, HR estimates available from the ACTT-1 trial [13] included in the meta-analysis were captured de facto in our sensitivity analysis because these HRs fell within the 95% CI of the PHD-informed HRs. Second, we did not evaluate the impact of changes in treatment practices other than an increase in evidence-based use of remdesivir. Although the increased potential lives saved in scenario 4 (remdesivir + DEX vs DEX monotherapy, Figure 1) highlight the potential benefit of remdesivir added to treatment regimen of patients receiving DEX alone, current NIH guidelines [7] recommend against use of DEX monotherapy in patients on “room air” (ie, NSOc patients). Adherence to this recommendation from the guidelines could potentially result in lives saved simply because of an “avoidance of harm”; the impact of this practice should be evaluated in future research. Third, although a PS matching approach [18–20] was used to control for implicit biases and for effect modification of remdesivir effectiveness, the effect of unmeasured confounders such as hybrid immunity or number of prior anti–COVID-19 immunizations was not explicitly considered since such data were not available in the PHD. It is possible that differences in factors such as vaccination status between RDV and no RDV groups could impact treatment effects or treatment decisions, thereby impacting the interpretation of the results. For the latter, it would be applicable if clinicians’ decisions on initiating remdesivir were based on vaccination status of a patient hospitalized for COVID-19. Finally, although there is evidence that the PHD is representative of US hospitalizations overall [6], its representativeness has not been formally evaluated. However, given the observed consistency in mortality risk across hospital characteristics, we do not believe adjustment to control for this uncertainty would meaningfully alter the results.

## CONCLUSION

The findings from this model underscore the potential value to public health of initiating remdesivir in accordance with current evidence-based best practices to minimize lives lost in patients hospitalized due to SARS-CoV-2 infection, regardless of supplemental oxygen requirements. Based on the emerging evidence, continuous reevaluation of institutional protocols for initiation of remdesivir in patients hospitalized for COVID-19 in the United States and potentially worldwide is therefore warranted, especially among vulnerable patients such as the elderly and immunocompromised.

## Supplementary Data

Supplementary materials are available at *Clinical Infectious Diseases* online. Consisting of data provided by the authors to benefit the reader, the posted materials are not copyedited and are the sole responsibility of the authors, so questions or comments should be addressed to the corresponding author.

## Supplementary Material

ciae517_Supplementary_Data

## Notes

***Author contributions.*** All authors contributed substantially to the manuscript and agreed to the final submitted version.

***Acknowledgments.*** Medical writing support was provided by Afrooz Afghani and Preetinder Kaur of Certara.

***Financial support*.** This work was supported by Gilead Sciences, Inc.

***Supplement sponsorship.*** This article appears as part of the supplement “Harnessing Real-world Data to Inform Clinical Practice: Lessons From the Evolving COVID-19 Era” sponsored by Gilead Sciences, Inc.

***Ethics approval and consent to participate.*** This study did not require ethics approval and informed consent. The analysis of data from the Premier Healthcare Database (US PINC AI Healthcare Database) was conducted under an exemption from the institutional review board oversight for US-based studies using deidentified healthcare records that are not reidentifiable.

***Availability of data and methods.*** The data supporting this study's findings are available from Premier, Inc (https://www.premierinc.com/). Restrictions apply to the availability of these data, which were used under license for this study.

***Potential conflicts of interest*.** A. C. K. reports grants from the National Institutes of Health Adaptive COVID-19 Treatment Trial. A. C., R. C., A. K., and M. Y. received funding and medical writing support from Gilead Sciences for conduct of this study. L. S. P. M. reports grants or contracts from National Institute for Health Research, CW + Charity, Infectopharm, LifeArc, North West London Pathology, Healthcare Infection Society, and Shionogi. He reports consulting fees from Umovis Lab and Pulmocide, payment or honoraria for lectures, presentations, speaker bureaus, manuscript writing or educational events from bioMerieux, Eumedica, Pfizer, and Shionogi. Additionally, he reports participation on data safety monitoring boards or advisory boards of Qiagen, bioMerieux, Shionogi, Sumiovant, Eumedica, Pfizer, and Gilead Sciences. He also has leadership or fiduciary roles in other board, society, committee or advocacy groups: Healthcare Infection Society, Middle East & North Africa Center for Research & Development and Internship, *Annals of Clinical Microbiology & Antimicrobials*, *Journal of Infection*, *Frontiers in Tropical Diseases*, *Nature Communications Medicine*, *Journal of Hospital Infection*, *Infectious Diseases*, *Microbiology, & Virology*, Chelsea & Westminster NHS Foundation Trust, Microbiology, North West London Pathology, Imperial College Healthcare NHS Trust, Imperial College London, and Clinical Infection Consulting Ltd. N. A. reports consulting fees from Gilead Sciences; payment or honoraria for lectures, presentations, speakers’ bureaus, manuscript writing, or educational events from Gilead Sciences and BMJ; support for attending meetings and/or travel from Gilead Sciences; and stock or stock options from Atropos. M. K. reports research grants from Pfizer; payment or honoraria for lectures from AstraZeneca, Berlin-Chemie, Boehringer Ingelheim, Gilead Sciences, GSK, Insmed, Pfizer; and participation in advisory boards for AstraZeneca, Sanofi, GSK, MSD, and Insmed; all outside of the submitted work. S. T. reports no conflict of interest. J. J. M. reports payment or honoraria for lectures, presentations, speaker bureaus, manuscript writing or educational events from Gilead Sciences and participation on the advisory boards of Gilead Sciences and AstraZeneca. E. M. is an employee and stockholder of Gilead Sciences. M. B. reports consulting fees and payment or honoraria for lectures, presentations, speaker bureaus, manuscript writing or educational events from Gilead Sciences, Pfizer, MSD, AdvanzPharma, AstraZeneca, Tillots, and Biomerieux. No other disclosures were reported.
