# Supplementary material for: Public Health Benefits of Applying Evidence-Based Best Practices in Managing Patients Hospitalized for COVID-19
Source: Clin Infect Dis. 2024 Oct 25;79(Suppl 4):S160–6. doi: 10.1093/cid/ciae517 (PMC11638767; doi:10.1093/cid/ciae517)
Supplement: ciae517_Supplementary_Data [file ciae517_supplementary_data.docx]

# Public Health Benefits of Applying Evidence-Based Best Practices in Managing Patients Hospitalized for COVID-19

Andre C Kalil^1^, Aastha Chandak^2^, Luke S.P. Moore^3^, Neera Ahuja^4^, Martin Kolditz^5^, Roman Casciano^2^, Ananth Kadambi^2^, Mohsen Yaghoubi^2^, Sotirios Tsiodras^6^, Jakob J. Malin^7^, Essy Mozaffari^8^, Michele Bartoletti^9-10^

*^1^Division of Infectious Diseases, University of Nebraska Medical Center, Omaha, Nebraska, USA*

*^2^Evidence and Access, Certara, New York, New York, USA*

*^3^Department of Infectious Diseases, Imperial College, London, United Kingdom*

*^4^Department of Internal Medicine, Stanford University School of Medicine, Stanford, California, USA*

*^5^Medical Department I, Division of Pulmonology, University Hospital Carl Gustav Carus, TU Dresden, Dresden, Germany*

*^6^Professor of Medicine and Infectious Diseases; National & Kapodistrian University of Athens Medical School; Chair, 4th Department of Internal Medicine, Attikon University Hospital, Athens, Greece*

*^7^Department I of Internal Medicine, Division of Infectious Diseases, Faculty of Medicine and University Hospital Cologne, University of Cologne, Cologne, Germany*

*^8^Medical Affairs, Gilead Sciences, Foster City, California, USA*

*^9^Department of Biomedical Sciences, Humanitas University, Via Rita Levi Montalcini 4, 20072 Pieve Emanuele, Milan, Italy*

*^10^Infectious Disease Unit, IRCCS Humanitas Research Hospital, Via Manzoni 56, Rozzano, 20089 Milan, Italy*

**Corresponding Author:**

Michele Bartoletti, MD, PhD

Associate Professor of Infectious Diseases,

Department of Biomedical Sciences

Humanitas University, Pieve Emanule (MI), Italy

Head of Infectious Diseases Unit

IRCCS Humanitas Research Hospital

Rozzano (MI), Italy

[michele.bartoletti@hunimed.eu](mailto:michele.bartoletti@hunimed.eu)

Tel. 0282243568

## SUPPLEMENTARY TABLES

### Supplementary Table 1. Hazard ratios* (95% CI) for the estimated 28-day in-hospital mortality benefit of initiation of remdesivir

| **Base case analysis: RDV treatment vs. No RDV treatment [1]** | | | |
| --- | --- | --- | --- |
|  | **HR** | **95% CI lower limit** | **95% CI upper limit** |
| NSOc | 0.71 | 0.61 | 0.83 |
| Any supplemental oxygen (LFO, HFO/NIV, or IMV/ECMO) | 0.79 | 0.70 | 0.88 |
| **Subgroup: Elderly (age ≥ 65 years) [1]** | | | |
|  | **HR** | **95% CI lower limit** | **95% CI upper limit** |
| NSOc | 0.71 | 0.60 | 0.84 |
| Any supplemental oxygen (LFO, HFO/NIV, or IMV/ECMO) | 0.72 | 0.64 | 0.82 |
| **Subgroup: Immunocompromised (IC) patients [2]✝** | | | |
|  | **HR** | **95% CI lower limit** | **95% CI upper limit** |
| NSOc | 0.90 | 0.67 | 1.22 |
| Any supplemental oxygen (LFO, HFO/NIV, or IMV/ECMO) | 0.74 | 0.58 | 0.94 |
| **Scenario 1: RDV treatment vs. No RDV treatment [1]** | | | |
|  | **HR** | **95% CI lower limit** | **95% CI upper limit** |
| NSOc | 0.77 | 0.72 | 0.83 |
| Any supplemental oxygen (LFO, HFO/NIV, or IMV/ECMO) | 0.79 | 0.74 | 0.83 |
| **Scenario 4: RDV+DEX combination vs. DEX monotherapy [3]** | | | |
|  | **HR** | **95% CI lower limit** | **95% CI upper limit** |
| NSOc | 0.80 | 0.74 | 0.88 |
| LFO | 0.74 | 0.68 | 0.81 |
| HFO/NIV | 0.71 | 0.65 | 0.78 |
| IMV/ECMO | 0.81 | 0.69 | 0.97 |

*HRs were derived using patient-level data across different periods of the Omicron-era SARS-CoV2 pandemic as follows: *late omicron period =* Jan 2023-Feb 2024 (used for base case and subgroups); *omicron-dominant period =* Dec 2021-Feb 2024 (used for Scenario 1); RDV + DEX combination vs. DEX monotherapy = Dec 2021-Apr 2023**.**

✝Note: HRs for immunocompromised (IC) patients were derived by applying previously published approach [2] to IC patient mortality data from the late omicron period.

Abbreviations: CI, confidence interval; DEX, dexamethasone; HFO/NIV, high-flow oxygen/non-invasive ventilation; HR, hazard ratio; IMV/ECMO, invasive mechanical ventilation/extracorporeal membrane oxygenation; LFO, low-flow oxygen; NSOc, no supplemental oxygen charges; RDV, remdesivir.

### Supplementary Table 2. Hazard ratios (95% CI) for the estimated 28-day in-hospital mortality benefit of initiating remdesivir in elderly patients in the Premier Healthcare Database cohort stratified by age*

| **Elderly (65-74 years)✝** | | | |
| --- | --- | --- | --- |
|  | **HR** | **95% CI lower limit** | **95% CI upper limit** |
| NSOc | 0.65 | 0.52 | 0.82 |
| Any supplemental oxygen (LFO, HFO/NIV, or IMV/ECMO) | 0.65 | 0.52 | 0.82 |
| **Elderly (75-84 years)✝** | | | |
|  | **HR** | **95% CI lower limit** | **95% CI upper limit** |
| NSOc | 0.80 | 0.68 | 0.94 |
| Any supplemental oxygen (LFO, HFO/NIV, or IMV/ECMO) | 0.80 | 0.68 | 0.94 |
| **Elderly (≥85 years)✝** | | | |
|  | **HR** | **95% CI lower limit** | **95% CI upper limit** |
| NSOc | 0.68 | 0.59 | 0.79 |
| Any supplemental oxygen (LFO, HFO/NIV, or IMV/ECMO) | 0.68 | 0.59 | 0.79 |

*Scenario 5 in the manuscript.

✝Note: The HRs for each elderly patient subgroup were derived by applying our previously reported methodology [1] to subsets of the Premier Healthcare Database patient-level data that cover only the late omicron period (Jan 2023-Feb 2024).

Abbreviations: CI, confidence interval; HFO/NIV, high-flow oxygen/non-invasive ventilation; HR, hazard ratio; IMV/ECMO, invasive mechanical ventilation/extracorporeal membrane oxygenation; LFO, low-flow oxygen; NSOc, no supplemental oxygen charges; PHD, PINC AI healthcare database.

### Supplementary Table 3. Demographics of the PHD population hospitalized for COVID-19 in 2023 by subgroup.

|  |  | **Immunocompromised (IC)** | **Elderly** | | **CCI ≥ 2** |
| --- | --- | --- | --- | --- | --- |
|  |  | **N=15116** | **N=65222** | | **N=53107** |
| **Age group (years)** | <18 | 213 (1.4%) | - | 117 (0.2%) | |
|  | 18-49 | 918 (6.1%) | - | 1962 (3.7%) | |
|  | 50-64 | 2,578 (17.1%) | - | 7,181 (13.5%) | |
|  | 65+ | 11,407 (75.5%) | 65,222 (100.0%) | 43,847 (82.6%) | |
|  | 65-74 | - | 18,440 (28.3%) | | - |
|  | 75-84 | - | 26,393 (40.5%) | | - |
|  | 85+ | - | 20,389 (31.3%) | | - |
| **Gender, Female** |  | 7,735 (51.2%) | 34,209 (52.5%) | | 26,680 (50.2%) |
| **Race** | White | 11,732 (77.6%) | 52,989 (81.2%) | | 40,968 (77.1%) |
|  | Black | 1,956 (12.9%) | 6,419 (9.8%) | | 7,125 (13.4%) |
|  | Asian | 390 (2.6%) | 1,740 (2.7%) | | 1,363 (2.6%) |
|  | Other | 1,038 (6.9%) | 4,074 (6.2%) | | 3,651 (6.9%) |
| **Ethnicity** | Hispanic | 1,428 (9.4%) | 4,861 (7.5%) | | 4,462 (8.4%) |
|  | Non-Hispanic | 12,755 (84.4%) | 56,286 (86.3%) | | 45,385 (85.5%) |
|  | Unknown | 933 (6.2%) | 4,075 (6.2%) | | 3,260 (6.1%) |
| **Hospital size, no. of beds** | < 100 | 1,289 (8.5%) | 7,041 (10.8%) | | 5,224 (9.8%) |
|  | 100-199 | 2,518 (16.7%) | 11,844 (18.2%) | | 9,102 (17.1%) |
|  | 200-299 | 2,685 (17.8%) | 12,472 (19.1%) | | 10,014 (18.9%) |
|  | 300-399 | 2,569 (17.0%) | 11,498 (17.6%) | | 9,359 (17.6%) |
|  | 400-499 | 1,834 (12.1%) | 7,436 (11.4%) | | 6,119 (11.5%) |
|  | 500+ | 4,221 (27.9%) | 14,931 (22.9%) | | 13,289 (25.0%) |
| **CCI** | 0 | 893 (5.9%) | 7,472 (11.5%) | | 0 (0.0%) |
|  | 1 | 1,696 (11.2%) | 13,903 (21.3%) | | 0 (0.0%) |
|  | 2 | 2,522 (16.7%) | 12,624 (19.4%) | | 15,691 (29.5%) |
|  | ≥ 3 | 10,005 (66.2%) | 31,223 (47.9%) | | 37,416 (70.5%) |
| **Comorbid conditions** | Obesity | 3,180 (21.0%) | 12,639 (19.4%) | | 13,068 (24.6%) |
|  | COPD | 5,836 (38.6%) | 23,373 (35.8%) | | 23,679 (44.6%) |
|  | Cardiovascular disease | 13,232 (87.5%) | 60,717 (93.1%) | | 50,591 (95.3%) |
|  | Diabetes | 5,335 (35.3%) | 24,799 (38.0%) | | 27,158 (51.1%) |
|  | Renal disease | 5,088 (33.7%) | 21,423 (32.8%) | | 24,933 (46.9%) |
|  | IC conditions^*^ | 15,116 (100.0%) | 11,407 (17.5%) | | 12,527 (23.6%) |
|  | Cancer | 6,681 (44.2%) | 5,408 (8.3%) | | 6,681 (12.6%) |
| **Baseline supplemental oxygen requirements** | NSOc | 10,065 (66.6%) | 43,921 (67.3%) | | 33,961 (63.9%) |
|  | LFO | 3,113 (20.6%) | 14,000 (21.5%) | | 11,442 (21.5%) |
|  | HFO/NIV | 1,665 (11.0%) | 6,366 (9.8%) | | 6,598 (12.4%) |
|  | IMV/ECMO | 273 (1.8%) | 935 (1.4%) | | 1,106 (2.1%) |
| **COVID-19 treatments upon admission** | Remdesivir+  Dexamethasone | 4,949 (32.7%) | 21,360 (32.7%) | | 17,334 (32.6%) |
|  | Dexamethasone monotherapy | 2,830 (18.7%) | 13,102 (20.1%) | | 11,050 (20.8%) |
|  | Remdesivir monotherapy | 2,986 (19.8%) | 10,669 (16.4%) | | 8,876 (16.7%) |
|  | Other monotherapy (baricitinib, tocilizumab, oral antivirals) | 223 (1.5%) | 1,083 (1.7%) | | 785 (1.5%) |
|  | None of the above COVID-19 treatments | 4,128 (27.3%) | 19,008 (29.1%) | | 15,062 (28.4%) |

Abbreviations: CCI, Charlson Comorbidity Index; COPD, chronic obstructive pulmonary disease; HFO/NIV, high-flow oxygen/non-invasive ventilation; HIV, human immunodeficiency virus; IC, immunocompromised; IMV/ECMO, invasive mechanical ventilation/extracorporeal membrane oxygenation; LFO, low-flow oxygen; NSOc, no supplemental oxygen charges.

*IC conditions include cancer, transplant, hematologic malignancies, immunosuppressive medications, toxic effects of antineoplastics, primary or severe combined immunodeficiencies, asplenia, bone marrow failure/aplastic anemia, or HIV.

### Supplementary Table 4. Potential lives saved in US during the omicron-dominant period, Dec 2021-Feb 2024

|  | Overall | Elderly  (age ≥ 65) | IC | CCI ≥ 2 |
| --- | --- | --- | --- | --- |
| PHD Population: N (95% CI) | | | | |
| Total potential lives saved with remdesivir treatment in the model cohort^*^ | **208**  (162-255) | **197**  (238-151) | **48**  (26-68) | **174**  (136-174) |
| NSOc | 87 (64-106) | 74 (53-95) | 18 (8-28) | 71 (87-52) |
| LFO | 64 (52-79) | 69 (55-80) | 17 (11-23) | 55 (44-68) |
| HFO/NIV | 56 (45-69) | 53 (43-62) | 12 (7-16) | 47 (38-59) |
| National-Level Projections: N (95% CI) | | | | |
| Total potential lives saved in the US in 2023 with additional remdesivir treatment | **731**  (570-898) | **595**  (457-718) | **253**  (140-361) | **786**  (614-967) |

*Patients in the model cohort were untreated patients that were PS-matched to remdesivir treated patients in the 2023 PHD cohort.

Abbreviations: CCI, Charlson Comorbidity Index; CI, confidence interval; HFO/NIV, high-flow oxygen/non-invasive ventilation; IC, immunocompromised; LFO, low-flow oxygen; NSOc, no supplemental oxygen charges; PHD, PINC AI healthcare database.

**Table S4** (above) presents the potential lives saved in 2023 if hazard ratios (HRs) from the omicron-dominant period of December 2021 to February 2024, were applied to the model cohort. The results are similar to the base case, with ~10% fewer lives saved in Scenario 1 (208 vs. 231, respectively). No supplemental oxygen charges (NSOc), low-flow oxygen (LFO), and high-flow oxygen/non-invasive ventilation (HFO/NIV) patients exhibited similar numbers of potential lives saved with remdesivir, although 95% confidence intervals (CIs) for the LFO and HFO/NIV groups in Scenario 1 differed from the base case, as the 95% CI for the late omicron and omicron-dominant period varied, while the point estimate of the HR remained the same.

## SUPPLEMENTARY FIGURES

### Supplementary Figure 1. Potential Lives Saved in 2023 by Initiating RDV in Model Cohort Elderly Patient Subgroups in the PHD^*`^


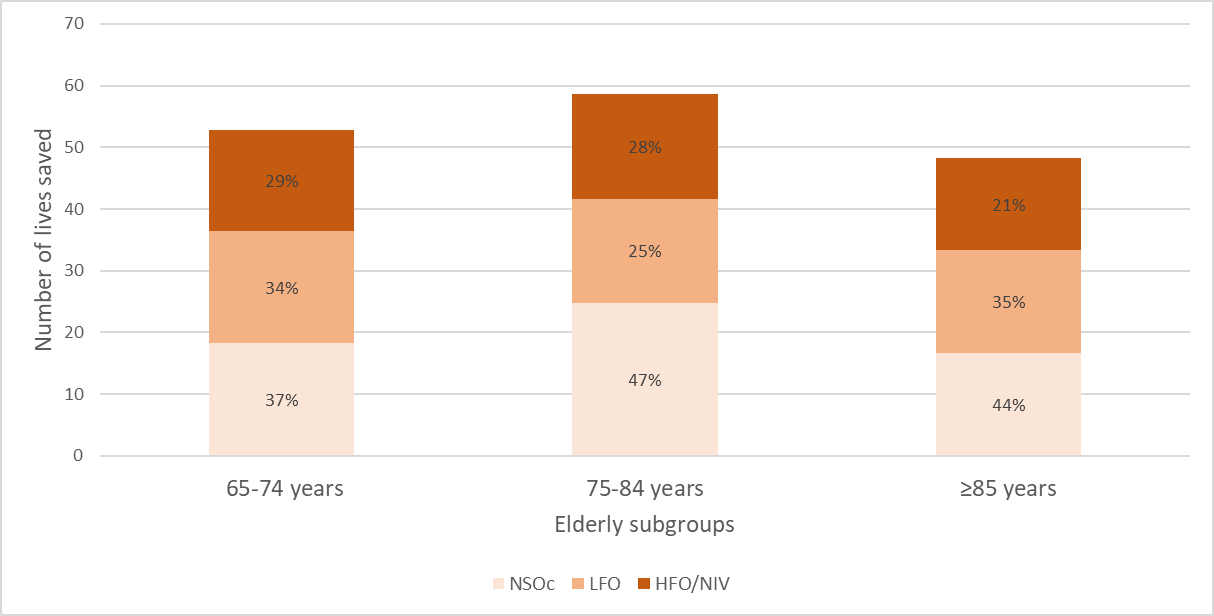


*Scenario 5 in the public health model.

Abbreviations: HFO/NIV, high-flow oxygen/non-invasive ventilation; LFO, low-flow oxygen; NSOc, no supplemental oxygen charges; PHD, PINC AI healthcare database; RDV, remdesivir.

## REFERENCES

1. Mozaffari E, Chandak A, Berry M, et al. Management of vulnerable patients hospitalized for Coronavirus Disease 2019 with remdesivir: a retrospective comparative effectiveness study of mortality in US hospitals. Clin Infect Dis 2024; 79:S137–48.
2. Mozaffari E, Chandak A, Gottlieb RL, et al. Remdesivir-associated survival outcomes among immunocompromised patients hospitalized for Coronavirus Disease 2019: real-world evidence from the Omicron dominant era. Clin Infect Dis 2024; 79:S149–59.
3. Mozaffari E, Chandak A, Gottlieb RL, et al. Lower mortality risk associated with remdesivir + dexamethasone versus dexamethasone alone for the treatment of patients hospitalized for COVID-19. Clin Infect Dis 2024:ciae477. doi: 10.1093/cid/ciae477. [Online ahead of print]
